# Association of loss of spleen visualization on whole-body diffusion-weighted imaging with prognosis and tumor burden in patients with multiple myeloma

**DOI:** 10.1038/s41598-021-03496-1

**Published:** 2021-12-14

**Authors:** Toshiki Terao, Youichi Machida, Ukihide Tateishi, Takafumi Tsushima, Kentaro Narita, Daisuke Ikeda, Ami Fukumoto, Ayumi Kuzume, Rikako Tabata, Daisuke Miura, Masami Takeuchi, Kosei Matsue

**Affiliations:** 1 Division of Hematology/Oncology, Department of Internal Medicine, Kameda Medical Center, 929 Higashi-chou, Kamogawa, 296-8602 Japan; 2 Department of Radiology, Kameda Medical Center, Kamogawa, Japan; 3 Department of Diagnostic Radiology, Tokyo Medical and Dental University, Tokyo, Japan

**Keywords:** Cancer imaging, Haematological cancer

## Abstract

This study investigated the clinical significance of loss of spleen visualization (LSV) on whole-body diffusion-weighted imaging (WB-DWI) in patients with multiple myeloma (MM). The WB-DWI of 96 patients with newly diagnosed MM (NDMM) and 15 patients with smoldering MM (sMM) were retrospectively reviewed. LSV was observed in 56 patients with NDMM (58.3%) and 1 patient with sMM (6.7%). Patients with NDMM with LSV had a higher median infiltration of bone marrow plasma cells (80.0% vs. 50.0%, *p* < 0.001) and median total diffusion volume (median; 540.2 vs. 137.0 mL, *p* = 0.003) than patients without LSV. Patients with LSV had a lower spleen-to-spinal cord ratio (0.36 vs. 0.96, *p* < 0.001) and worse 2-year overall survival (OS) (84.6% vs. 100%, *p* = 0.032). Patients who did not recover spleen visualization during treatment had a worse prognosis, even when they obtained very good partial response (median progression-free survival: 13.2 months). Spleen histopathological findings revealed higher cellularity and diffuse myeloma cell infiltration in a patient with LSV and splenic amyloidosis without extramedullary hematopoiesis in a patient without LSV. Therefore, LSV indicates worse prognosis for patients with MM, even when the patient responds to treatment. Further studies are warranted to clarify the immunological role of spleen in MM.

## Introduction

Multiple myeloma (MM) is caused by the proliferation of monoclonal malignant plasma cells (PCs) in the bone marrow (BM). Positron-emission tomography/computed tomography (PET/CT) and whole-body diffusion-weighted imaging (WB-DWI) on magnetic resonance imaging (MRI) have been used in patients with newly diagnosed MM (NDMM)^1–3^. DWI contrast is generated from differences in water molecule mobility between the tissue environments. Highly cellular tissues such as tumors (where water diffusion is restricted), or tissues where the proportion of water density is higher than cellular density (such as in a cerebral infarction), result in high signal intensity on DWI with high *b*-values compared to the normal surrounding tissue. In patients with MM, the use of WB-DWI in diagnosis is superior to whole-body radiographs^4^ and is equal to or more sensitive than PET/CT for the detection of focal lesions^1,5,6^. Although WB-DWI is mainly used in diagnostic process, there are several reports regarding the use of WB-DWI to monitor the patient’s response to treatment^7–9^. Myeloma Response Assessment and Diagnosis System (MY-RADS) recommendation was designed to report WB-MRI and assess treatment response^8^. Moreover, recently, patients who achieved the complete imaging response after autologous stem-cell transplantation (ASCT) showed significant superior survival than those with imaging residual disease^9^.

We previously reported that the total diffusion volume (tDV) by pre-treatment WB-DWI was associated with a high bone marrow plasma cell (BMPC) count and poor prognosis in patients with MM^10^. Tumor volumes detected by WB-DWI and PET/CT were correlated, and we unexpectedly frequently found absence of a spleen signal in patients with NDMM and its reappearance after treatment. Rasche et al. reported that lack of spleen visualization on WB-DWI is related to a high tumor burden and poor prognosis^11^. The previous study from Rasche et al. suggested that extramedullary hematopoiesis (EMH) in the spleen resulted in the loss of the spleen signal using Tc-99 m-labeled anti-CD66.

This study aimed to investigate the association between WB-DWI, myeloma load and prognosis in patients with MM, focusing on: (1) the relationship among spleen signal loss, tDV, and tumor volume at diagnosis; (2) the change in spleen signal intensity and visualization during treatment; (3) the relationship between follow-up spleen signal, follow-up tDV, clinical data, and prognosis; and (4) the mechanism of the loss of spleen signal.

## Methods

### Study design and patients

The data of 96 consecutive patients with symptomatic NDMM diagnosed at Kameda Medical Center from January 2016 to December 2020, 15 consecutive patients with smoldering MM (sMM), and 2 autopsied spleens of patients with PC dysclasia (1 monoclonal gammopathy of undetermined significance and 1 primary plasma cell leukemia) were retrospectively reviewed. The diagnosis and treatment responses of patients with MM were assessed using the International Myeloma Working Group (IMWG) criteria^12,13^. The observation period was completed on October 30, 2021. All patients underwent at least one WB-DWI prior to treatment. This study was approved by the institutional review board of Kameda Medical Center (No. 20–135) and conducted in accordance with the Declaration of Helsinki. All participants provided informed consent.

### Cytogenetic analysis

High-risk cytogenetic abnormalities (CAs) were del(17p), t(4;14), or t(14;16) using interphase fluorescence in situ hybridization (iFISH) analysis. A gain of 1q21 was also investigated in all patients except for 2 with symptomatic MM. The iFISH analyses were performed prior to treatment using PCs purified with CD138-coated magnetic beads (Miltenyi Biotec, Bergisch Gladbach, Germany). The cutoff values of del(17p) and gain of 1q21 were 10% and 20%, respectively^14,15^.

### Acquisition and analysis of magnetic resonance images

As described in our previous report^10^, WB-DWI were obtained using a 1.5-Tesla unit (Magnetom Vision; Siemens Healthcare, Germany) with the following parameters: acquisition type, 2D; repetition time, 5400 ms; echo time, 74 ms; inversion time, 180 ms; slice thickness, 5 mm; and *b*-values, 0 and 900 s/mm^2^. The apparent diffusion coefficient (ADC) was calculated voxel by voxel for each image slice in *b* = 0 and 900 images. The tDV was calculated using regions of interest (ROIs) that included areas of abnormally high DWI signal intensity, which contained myeloma lesions in the BM or extramedullary tissue. The ROIs were automatically obtained using medical imaging software (BD Score; PixSpace, Japan)^16^.

**Figure 1:**
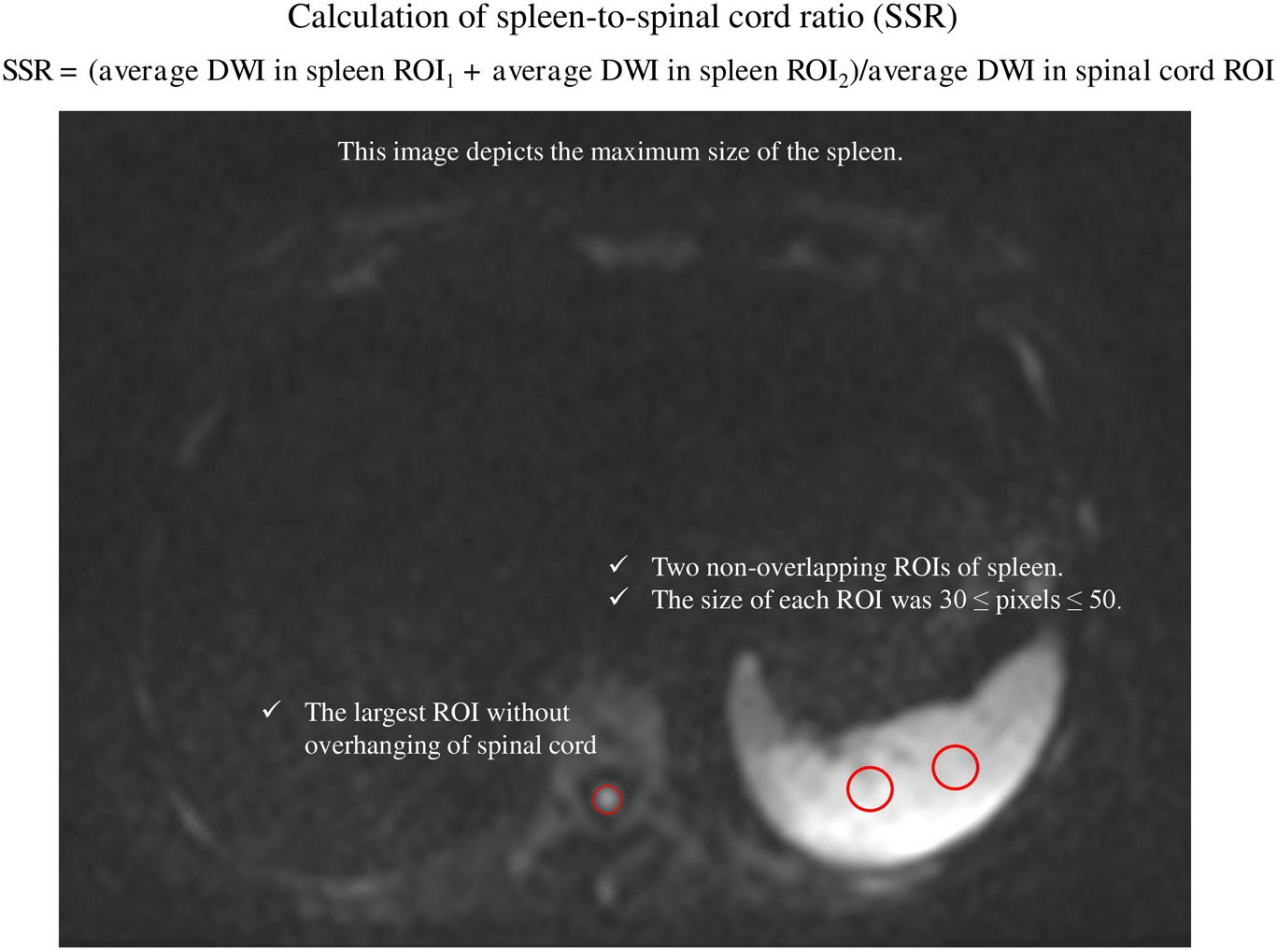
Calculation of spleen-to-spinal cord ratio. The spleen-to-spinal cord ratio (SSR) is calculated as the sum of the average diffusion-weighted imaging (DWI) in spleen (region of interest) ROI_1_ and spleen ROI_2_ divided by the average DWI in the spinal cord ROI.

Loss of spleen visualization (LSV) was defined as a visual loss of the spleen in maximum intensity projection on the WB-DWI. The spleen-to-spinal cord (SC) ratio (SSR) was calculated using the ratio of the signal intensity of the spleen and SC. The calculations are shown in Fig. 1. The spleen ROIs were defined as nonoverlapping ROIs of 30–50 pixels in *b* = 900 images. The SC ROI was the largest ROI without overhangings in the image depicting the maximum size of the spleen^17^. The SC served as an in vivo reference as it had a higher cellular density and lower mitosis rate, was less affected by blood transfusions and anti-myeloma medications, and was less likely to be infiltrated with myeloma cells at diagnosis and during follow-up. Moreover, SC constantly acquired with reasonably limited diffusion unless affected by neurologic pathology^17^.

### Statistical analysis

The Kaplan–Meier method with a log-rank test was used for the survival analyses. Progression-free survival (PFS) and overall survival (OS) were measured from diagnosis to relapse or death from any cause or censored at the date of last contact. The Wilcoxon rank sum test, Kruskal–Wallis rank sum test, and Fisher’s exact test were used to compare the medians of continuous variables or the distributions of categorical variables. All statistical analyses were performed using EZR software (Saitama Medical Centre, Jichi Medical University, Japan)^18^. Statistical significance was set at *P* < 0.05.

### Ethics approval

All procedures performed in studies involving human participants were in accordance with the ethical standards of the institutional and/or national research committee and with the 1964 Helsinki Declaration and its later amendments or comparable ethical standards. The study was approved by the institutional review board of Kameda Medical Center (No. 20–135).

### Consent to participate

Informed consent was obtained from all individual participants included in the study.

### Consent for publication

Patients signed informed consent regarding publishing their data and photographs.

## Results

### Patient characteristics

The baseline clinical characteristics of patients with symptomatic NDMM are summarized in Table 1. The median patient age was 75.5 years (range: 30–89 years), and 81 patients (84.4%) were 65 years or older. The median follow-up period was 29.5 months (range; 0.2–70.7 months). Twenty-two patients (24.0%) had high-risk CAs, 34 (36.2%) had a gain of 1q21, 48 (50.0%) were international staging system (ISS) stage III, and 22 (24.0%) were revised-ISS (R-ISS) stage III. Disease progression was observed in 33 patients (34.4%), and 17 patients (17.7%) died. Almost all patients (n = 91, 94.8%) received proteasome inhibitors as remission induction therapy, and 69 patients (71.9%) received a triple induction regimen, including 50 patients who received bortezomib, lenalidomide, and dexamethasone. Thirty-two patients (33.3%) received ASCT. The median PFS was 50.4 months, and the 2-year PFS was 74.8% (95% confidence interval [CI] 64.1–82.7%). The median OS was not reached, and the 2-year OS was 91.0% (95% CI 82.7–95.4%).

**Table 1:** Patient characteristics and differences by spleen signal.

Characteristics	All	w/o LSV	LSV	*P*
	N = 96	n = 40	n = 56	
Age, years [median (range)]	75.5 (30, 89)	76 (30, 89)	73 (50, 87)	0.21
Sex, male (%)	46 (47.9)	16 (40.0)	30 (53.6)	0.65
Albumin, g/dL [median (range)]	3.3 (1.6, 5.0)	3.6 (1.7, 5.0)	2.9 (1.6 4.7)	0.001
Beta 2-microglobulin, mg/L [median (range)]	5.1 (1.5, 54.2)	3.1 (1.6, 16.7)	6.0 (1.5, 54.2)	< 0.001
Calcium, mg/dL [median (range)]	9.8 (8.3, 15.4)	9.3 (8.5, 13.5)	10.0 (8.3, 15.4)	< 0.001
Creatinine, mg/dL [median (range)]	0.95 (0.40, 10.5)	0.80 (0.50, 9.2)	1.10 (0.40, 10.5)	0.005
Haemoglobin, g/dL [median (range)]	9.6 (5.4, 15.5)	10.2 (6.6, 15.5)	9.2 (5.4, 15.0)	0.004
Heavy-chain type, IgG, (%)	53 (55.2)	25 (62.5)	28 (50.0)	0.3
Light-chain type, kappa, (%)	61 (64.2)	21 (52.5)	42 (72.7)	0.053
LDH, high (%)	28 (29.2)	7 (17.5)	21 (37.5)	0.041
High-risk CAs (%)^a^	22 (24.0)	7 (17.5)	16 (28.5)	0.24
Gain of 1q21 (%)*^a^	34 (36.2)	11 (27.5)	23 (42.6)	0.19
ISS, stage III (%)	48 (50.0)	13 (32.5)	35 (62.5)	0.007
R-ISS, stage III (%)	22 (22.9)	3 (7.5)	19 (33.9)	0.003
BM plasma cells by CD138, % [median (range)]	68.9 (1.2, 100)	50.0 (13.5, 100)	80.0 (1.2, 100)	< 0.001
Induction, triplet (%)	69 (71.9)	28 (70.0)	41 (73.2)	0.77
Induction, with PIs (%)	91 (94.8)	37 (92.5)	54 (96.4)	0.65
ASCT recipients (%)	32 (33.3)	11 (27.5)	21 (37.5)	0.38
FDG-uptake in spleen in PET/CT # (%)	6 (6.5)	1 (2.6)	5 (9.4)	0.23
tDV, mL [median (range)] §	289.5 (3.1, 1837.6)	137.0 (3.1, 1387.9)	540.2 (5.9, 1837.6)	0.003
SSR, [median (range)]	0.54 (0.16, 2.64)	0.96 (0.44, 2.64)	0.36 (0.16, 1.19)	< 0.001

*n = 94.

^#^n = 92.

^§^ n = 90.

^a^The cut-off value of del(17p) and gain of 1q21 were 10% and 20%, respectively.

Abbreviations: ASCT; autologous stem-cell transplantation, BM; bone marrow, CAs; cytogenetic abnormalities, FDG; fluorodeoxyglucose, IgG; immunoglobulin G, ISS; International Staging System, LDH; lactate dehydrogenase, LSV; loss of spleen visualization, MM; multiple myeloma, PET/CT; positron-emission tomography/computed tomography, PIs; proteasome inhibitors, R-ISS; revised-International Staging System, SSR; spleen-to-spinal cord ratio.

### Associations between loss of spleen visualization, spleen-to-spinal cord ratio, and patient characteristics

**Figure 2:**
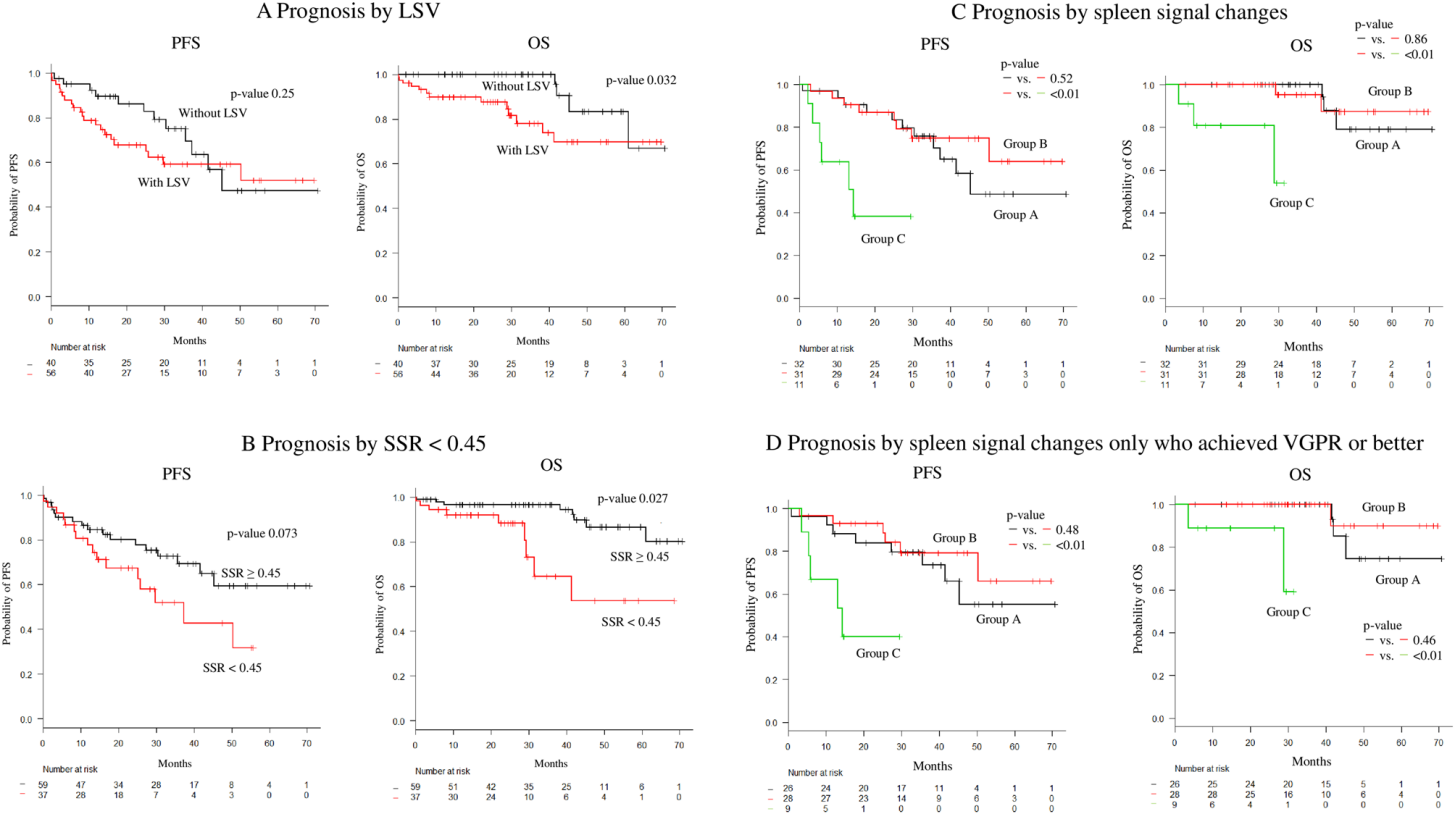
Survival impact of the absent spleen sign on whole-body diffusion-weighted imaging. (**a**) Patients with LSV showed worse 2-y PFS and 2-y OS than those without LSV (2-y PFS; 66.9% vs. 86.0%, *P* = 0.25, and 2-y OS; 84.6% vs. 100%, *P* = 0.032). (**b**) Patients with a spleen-to-spinal cord ratio (SSR) < 0.45 have worse prognoses than those with an SSR ≥ 0.45 (2-y PFS; 67.6% vs. 79.4%, *P* = 0.073, and 2-y OS; 85.1% vs. 94.6%, *P* = 0.027). (**c**) Patients with loss of spleen visualization (LSV) at diagnosis and during follow-up despite treatment response (group C) have a significantly worse prognosis than those who do not have LSV (group A) and those who had LSV at diagnosis but in whom the spleen reappears after a favorable treatment response (group B). (**d**) Upon exclusion of patients with a poor treatment response (n = 11), group C (n = 9) continues to have a worse prognosis than that of groups A and B. PFS: progression-free survival; OS: overall survival; VGPR: very good partial response.

LSV was observed on the WB-DWI of 56/96 (58.3%) patients with NDMM (Table 1) and in 1 patient with sMM (1/15, 6.7%). Patients with NDMM and LSV had higher median BMPC infiltration compared to those without LSV as assessed by CD138-immunohistochemistry (80.0% vs. 50.0%, *P* < 0.001), a higher median tDV (540.2 mL vs. 137.0 mL, *P* = 0.003), lower hemoglobin levels (*P* < 0.01), higher creatinine levels (*P* < 0.01), and a higher rate of ISS stage III (*P* < 0.01). The 2-year PFS and 2-year OS were lower in patients with NDMM with LSV (PFS: 66.9%, 95% CI 52.0–78.1%; OS: 84.6%, 95% CI 71.4–92.1%) than in patients without LSV (PFS: 86.0%, 95% CI 69.3–94.0%, *P* = 0.25; OS: 100%, 95% CI 100–100%, *P* = 0.032) (Fig. 2a).

The correlations between ^18^F-fluorodeoxyglucose (^18^F-FDG) uptake in the spleen and LSV were investigated in 92 patients (95.8%) who underwent WB-DWI and PET/CT simultaneously during the initial diagnostic evaluation. LSV was observed in 53 (57.6%) patients, while 6 patients (6.5%) had higher ^18^F-FDG spleen uptake than liver (equivalent to 4 or more in the Deauville 5-point scale^19^), 5 of whom had LSV (Table 1).

The median SSR in patients with NDMM (0.54; range: 0.16–2.64) was lower than that in patients with sMM (1.56; range: 0.37–3.72) (*P* < 0.001). Patients with NDMM with LSV had a lower median SSR (0.36 vs. 0.96; *P* < 0.001) and tDV (540.2 vs. 137.0 mL; *P* = 0.003) than those without LSV (Supplemental Fig. S1a, b). Patients with ISS stage I and R-ISS stage I had a higher SSR than those with ISS stage II-III or R-ISS stage II–III, respectively (Supplemental Fig. S1c, d). The SSR was negatively correlated with BMPC infiltration (Spearman’s coefficient [ɤs] = -0.43; *P* < 0.001) and beta-2 microglobulin (ɤs = − 0.39, *P* < 0.001) (Supplemental Fig. S1e, f.).

The SSR cutoff value for predicting the disease progression was 0.45 (area under the curve: 0.551), as estimated using receiver operating characteristic curves. Patients with an SSR < 0.45 (n = 37) had worse 2-year PFS compared to those with an SSR ≥ 0.45 (67.6%, 95% CI 48.8–80.8%, vs. 79.4%, 95% CI 65.7–88.1%; *P* = 0.073) and 2-year OS (85.1%, 95% CI 67.5–93.6%, vs. 94.6%, 95% CI 84.2–98.2%; *P* = 0.027) (Fig. 2b). There was no significant difference in tumor characteristics such as heavy-chain type (IgG or others), light-chain type (kappa or lambda), high-risk CAs, and gain of 1q21 between patients with high and low SSR.

### Prognostic impact of spleen signal changes during treatment

The spleen signal change was evaluated in 74 (77.1%) patients with NDMM who underwent WB-DWI at least twice (before and during treatment) (Table 2). The median follow-up period in these 74 patients was 33.8 months (range; 3.6–70.7 months). Although WB-DWI was performed at each physician’s discretion, it was generally performed when patients achieved very good partial response (VGPR) or better. The median SSR of patients with an SSR < 0.45 at diagnosis (n = 29, 39.2%) increased to 0.62 on the follow-up WB-DWI.

**Table 2:** Patient characteristics based on spleen signal changes.

Characteristics	Group A	Group B	Group C	*P*
	n = 32	n = 31	n = 11	
Age, years [median (range)]	75 (30, 89)	71 (50, 87)	74 (52, 80)	0.37
Sex, male (%)	12 (37.5)	16 (51.6)	5 (45.5)	0.53
Albumin, g/dL [median (range)]	3.6 (1.7, 5.0)	3.2 (2.1, 4.5)	2.7 (1.8, 3.7)	0.011
Beta 2-microglobulin, mg/L [median (range)]	3.1 (1.6, 13.4)	5.9 (1.5, 34.6)	10.2 (2.5, 50.3)	0.003
Calcium, mg/dL [median (range)]	9.3 (8.5, 13.5)	9.8 (8.4, 13.4)	10.0 (8.3, 15.4)	0.031
Creatinine, mg/dL [median (range)]	0.8 (0.5, 9.2)	1.0 (0.4, 10.5)	1.5 (0.6, 9.9)	0.049
Hemoglobin, g/dL [median (range)]	10.1 (6.6, 15.5)	9.4 (5.4, 15.0)	8.5 (6.5, 13.5)	0.06
Heavy-chain type, IgG, (%)	18 (56.2)	13 (41.9)	6 (54.5)	0.5
Light-chain type, kappa, (%)	19 (59.4)	21 (70.0)	9 (81.8)	0.36
LDH, high (%)	6 (18.8)	12 (28.7)	5 (45.5)	0.12
High-risk CAs (%)	6 (18.8)	5 (16.1)	6 (54.5)	0.025
Gain of 1q21 (%)*	9 (28.1)	12 (40.0)	4 (40.0)	0.58
ISS, stage III (%)	10 (31.2)	20 (64.5)	8 (72.7)	0.009
R-ISS, stage III (%)	2 (6.2)	9 (29.0)	7 (63.6)	< 0.001
BM plasma cells by CD138, % [median (range)]	45.4 (13.5, 90.0)	72.5 (5.0, 100.0)	95.0 (1.2, 100)	< 0.001
Induction, triplet (%)	23 (71.9)	25 (80.6)	7 (63.6)	0.52
Induction, with PIs (%)	30 (93.8)	30 (96.8)	11 (100.0)	0.63
ASCT recipients (%)	11 (34.4)	16 (51.6)	3 (27.3)	0.24
Median follow-up WB-DWI, times, [median (range)]	3 (2, 8)	3 (2, 7)	3 (2, 12)	0.42
Period from diagnosis to follow-up WB-DWI, months, [median (range)]	19.4 (2.7, 58.1)	11.9 (2.5, 41.0)	5.1 (1.4, 28.3)	0.001
**Treatment response at follow-up WB-DWI**				0.22
sCR	19 (59.4)	22 (71.0)	3 (27.3)	
CR	1 (3.1)	3 (9.7)	2 (18.2)	
VGPR	6 (18.8)	3 (9.7)	4 (36.4)	
PR	2 (6.2)	2 (6.5)	2 (18.2)	
SD	1 (3.1)	0 (0.0)	0 (0.0)	
PD	3 (9.4)	1 (3.2)	0 (0.0)	
Follow-up tDV at best response, mL [median (range)]§	64.3 (1.6, 373.3)	43.5 (1.6, 312.9)	58.5 (6.1, 374.1)	0.65
Follow-up SSR at best response, [median (range)]	1.49 (0.32, 2.62)	1.35 (0.65, 2.85)	0.38 (0.16, 0.97)	< 0.001

*n = 72.

^§^n = 69.

Abbreviations: ASCT, autologous stem-cell transplantation, BM; bone marrow, CAs; cytogenetic abnormalities, CR; complete response, IgG; immunoglobulin G, ISS; International Staging System, LDH; lactate dehydrogenase, PD; progressive disease, PIs; proteasome inhibitors, PR; partial response, R-ISS; revised-International Staging System, sCR; stringent complete response, SD; stable disease, SSR; spleen-to-spinal cord ratio, VGPR; very good partial response, WB-DWI; whole-body diffusion-weighted imaging.

**Figure 3:**
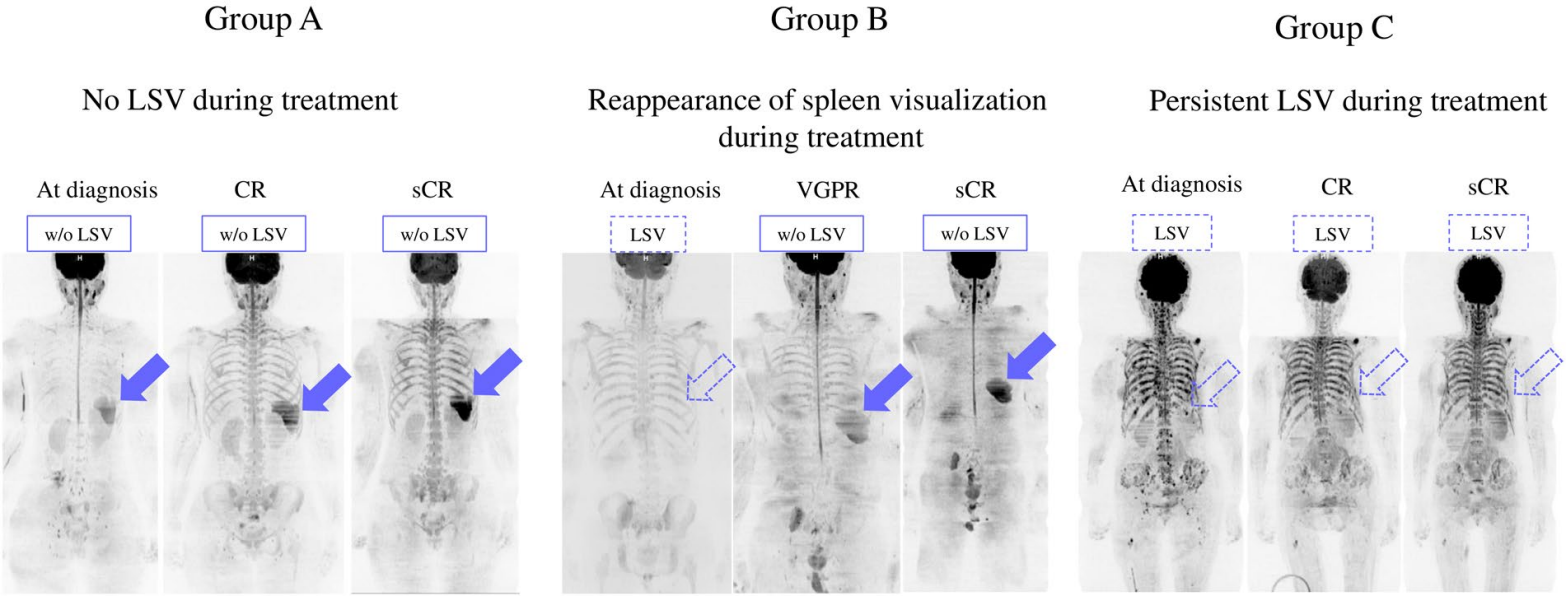
Representative patients with spleen signal changes in whole-body diffusion-weighted imaging. (**a**) Group A: Patients without loss of spleen visualization (LSV) at diagnosis or during treatment. (**b**) Group B: Patients with LSV at diagnosis but who have a visible spleen during treatment. (**c**) Group C: Patients who have LSV at diagnosis and during treatment despite a favorable treatment response. Blue arrows indicate visible spleen. Arrows outlined in blue indicate invisible spleen. CR: complete response; VGPR: very good partial response; w/o: without.

Of 42 out of the 74 patients with LSV at diagnosis, the spleen became visible on 31/42 (73.8%) patients during treatment. Representative patients with various spleen signal changes during treatment are shown in Fig. 3 as group A (n = 32; patients without LSV at diagnosis and during treatment), group B (n = 31; patients with LSV at diagnosis but whose spleen reappeared after treatment), and group C (n = 11; patients with LSV at diagnosis and, despite treatment response, did not regain the spleen signal) (Table 2).

The induction regimens and treatment responses of groups A, B, and C were not significantly different. The proportion of patients who received ASCT was relatively lower in group C, and these patients had a shorter duration until the follow-up WB-DWI (5.1 months) compared with the patients in group A (19.4 months) and B (11.9 months) (*P* = 0.001). Patients in group C had a significantly worse 2-year PFS (38.2%; 95% CI 9.9–67.0%) than those in group A (86.9%; 95% CI 68.8–94.9%; *P* = 0.003) and group B (87.0%; 95% CI 68.9–94.9%; *P* = 0.003). The 2-year PFS was not significantly different between group A and group B (*P* = 0.52). Patients in group C had a significantly worse 2-year OS (80.8%, 95% CI 42.3–94.9%) than those in group A (100.0%; 95% CI 100–100%; *P* = 0.001) and group B (95.2; 95% CI: 70.7–99.3%; *P* = 0.004). The 2-year OS was not significantly different between groups A and B (*P* = 0.86) (Fig. 2c).

Additional analyses were conducted after excluding patients who achieved a poor response (n = 11). Overall, 63 patients responded to treatment with VGPR or better. These patients had a median follow-up tDV of 42.3 mL and a reduction of 246.7 mL from the median tDV at diagnosis (*P* < 0.001). The median PFS in group C (n = 9) were 14.3 (range: 3.6 months–not available). The 2-year PFS was worse in group C (40.0%; 95% CI 9.8–69.7%) than in group A (83.9%; 95% CI 62.6–93.7%; *P* = 0.014) and in group B (92.9%; 95% CI 74.3–98.2%; *P* = 0.004). The 2-year PFS was not significantly different between groups A and B (*P* = 0.48). Patients in group C had a significantly worse 2-year OS (88.9%; 95% CI 43.3–98.4%) than those in group A (100.0%; 95% CI: 100–100%; *P* = 0.01) and group B (100.0%; 95% CI 100–100%; *P* = 0.01). The 2-year OS was not significantly different between groups A and B (*P* = 0.46) (Fig. 2d).

### Histopathological findings

We reviewed two patients who had both received WB-DWI within 3 months before their deaths. One patient who had primary plasma cell leukemia with LSV died due to disease progression and had diffuse myeloma cell infiltration in the spleen (Supplemental Fig. S2a, c). The other patient who had a monoclonal gammopathy of undetermined significance with systemic light-chain amyloidosis without LSV died due to cardiac amyloidosis and sepsis (Supplemental Fig. S2) and had amyloid deposition in the spleen, but no myeloma cell infiltration (Supplemental Fig. S2b, d). No evidence of EMH in the spleen was detected in any patient using CD34, CD42b, or factor VIII immunohistochemistry (the CD34 immunohistochemistry results are shown in Supplemental Fig. S2e, f).

## Discussion

In this study, LSV was associated with a high tumor burden and poor prognosis in patients with MM. Patients with LSV had a lower SSR and a negative correlation between the SSR and beta-2 microglobulin or BMPC was also identified, suggesting that SSR inversely reflected the myeloma burden. Visualization of the spleen during treatment was not restored in some patients (11/74, 14.9%), moreover, they had extremely worse outcomes despite achieving favorable treatment response.

Rasche et al. reported that the lack of a spleen signal was associated with a higher tumor volume and poorer prognosis, which is consistent with the results of the current study^11^. However, there was a difference in the percentage of patients with of spleen loss in our report (58.3%) and in the previous reports (24%). The current study included more older patients (median age: 75.5 years) and more advanced-stage MM (50.0% in ISS stage III) than the previous study (26% in ISS stage III). Moreover, our definition of LSV was looser than that of the previous report by Rasche. These differences in the patients characteristics and the spleen loss definition might have contributed to the difference in the percentage of patients with spleen loss between our report and in the previous report.

The median tDV decreased in patients with LSV who responded to treatment. Typically, the tDV increases in lesions with high cellularity and in patients with lesions that have an increased ADC value, such as in those with edema of the BM cells during hematopoietic recovery^20^ or with changes in the microenvironment of the BM due to hematopoietic stimulating factor use. LSV may be useful in differentiating such reactive signaling from enhancement due to tumor invasion. In this study, ADC thresholds were obtained at 0 and 900 × 10^–3^ mm^2^/s. However, it may be necessary to change the ADC thresholds to assess the tDV after treatment, as described in the MY-RADS^8^

While LSV is easy to evaluate and is clinically valuable, the mechanism of LSV on WB-DWI remains unknown. Rasche et al. speculated that LSV is due to EMH in the spleen as anti-CD66 monoclonal antibodies were diffusely positive in the spleen of a patient with a large residual myeloma burden^11^. CD66 was reported to be positive in myeloid cells including promyelocytes and granulocytes^21,22^ and was used as an evidence of EMH in a patient with myelofibrosis^23^. However, CD66 has been reported to be positive by flow cytometry analysis in approximately three quarters, but not all the patients with myeloma^24–26^. We found a patient with sMM complicated with myeloproliferative disorder who had a diffuse BM signal on WB-DWI but did not have LSV, although EMH was present (Supplemental Fig. S3). In addition, no pathological EMH was observed in the two autopsied spleens. ^18^F-FDG uptake by the spleen can be observed on PET/CT scans in most patients with myeloproliferative disorders who have EMH^27^; however, ^18^F-FDG uptake at diagnosis in the spleen than in the liver was observed in only six patients with NDMM in this study despite approximately half of patients with NDMM having LSV on WB-DWI. Furthermore, spleen EMH in hematologic malignancies typically presents as a nodular, not diffuse, pattern^28^. Based on our findings and those of previous reports, the cause of LSV might not be due to EMH in the spleen.

The microenvironment of the spleen in patients with MM is largely unknown. The spleen is the organ of antibody production and some normal PCs are found in the spleen^29^. Indeed, Shapiro et al. reported that there was an increase of the percentage of myeloma cells in the splenic aspirations comparable to that in the marrow^30^. In the previous report, only 3/10 of the patients had palpable spleen. Although we considered the possibility that myeloma cells might have escaped from the BM niche into the spleen in the patients with LSV, there were no findings to support this. However, due to the poor prognosis of patients who had LSV at diagnosis and even at favorable treatment response, they should be evaluated by other methods such as measurable residual disease and be considered for change to other treatment regimens.

WB-DWI is a more feasible imaging technique than PET/CT as it is more cost-effective, less time-consuming (up to 30 min), requires no breath-holding, and does not expose the patient to radiation^31,32^. WB-MRI is currently used to detect focal lesions and determine the indication of treatment according to the IMWG criteria^12^. Moreover, recent studies demonstrated the efficacy of repetitive WB-MRI for treatment response and prognostic impact^7–9^. LSV, which could be additionally evaluated in the WB-DWI of diagnostic process, could become an additional and simplified prognostic indicator in patients with NDMM.

On the other hand, several hospitals are unable to perform WB-MRI. For such institutions, the IMWG consensus statement suggested the spine or pelvic MRI instead of WB-MRI^33^. In the current criteria, these MRI (WB-MRI or spinal/pelvic MRI) could only be used to evaluate treatment initiation criteria, and not used to evaluate the tumor load. Our SSR was simple to obtain (imaging in the spleen slice in *b* = 900 images) and could help the tumor load assessment. Moreover, in the hospitals where WB-MRI was not available, the repetitive SSR assessment could help detect patients with poor prognosis, such as those who never regain the spleen visualization (group C).

This study is not without limitations, including its retrospective nature, small sample size, and relatively short observation period. Multivariate analysis of group C in 63 patients who achieved VGPR or better could not be performed owing to the small number of events (19 progression and 6 death). The spleen pathology and WB-DWI could not be compared in most patients, as patients with MM rarely received spleen biopsy due to the risk of bleeding. While the direct infiltration of myeloma cells into the spleen has been proposed as a mechanism of LSV, the small patient population in this study could not provide enough power to verify this hypothesis. Moreover, we did not evaluate T1- or T2-weighted imaging or the potential risk of false positives or negatives of LSV. Our LSV and the “lack of a spleen signal” in the Rasche’s report detected almost the same phenomenon; however, the MRI conditions or various parameters were slightly different.

In conclusion, this study showed that LSV and a low SSR on pre-treatment WB-DWI are correlated with high tumor volume and poor prognosis. As patients with LSV during treatment have very poor prognosis despite favorable treatment responses, the relationships between LSV and other variables should be investigated. The mechanism of LSV in WB-DWI remains unknown. Future studies that include pathological and microenvironment analysis of the spleen are necessary to validate our results, and to determine the unique function of the spleen in patients with MM.

## Supplementary Information

Supplementary Information 1.

Supplementary Information 2.

Supplementary Information 3.

Supplementary Information 4.

## Data Availability

The datasets generated during and/or analysed during the current study are available from the corresponding author on reasonable request.
