# Supplementary figures and images for: Association of loss of spleen visualization on whole-body diffusion-weighted imaging with prognosis and tumor burden in patients with multiple myeloma
Source: Sci Rep. 2021 Dec 14;11:23978. doi: 10.1038/s41598-021-03496-1 (PMC8671425; doi:10.1038/s41598-021-03496-1)

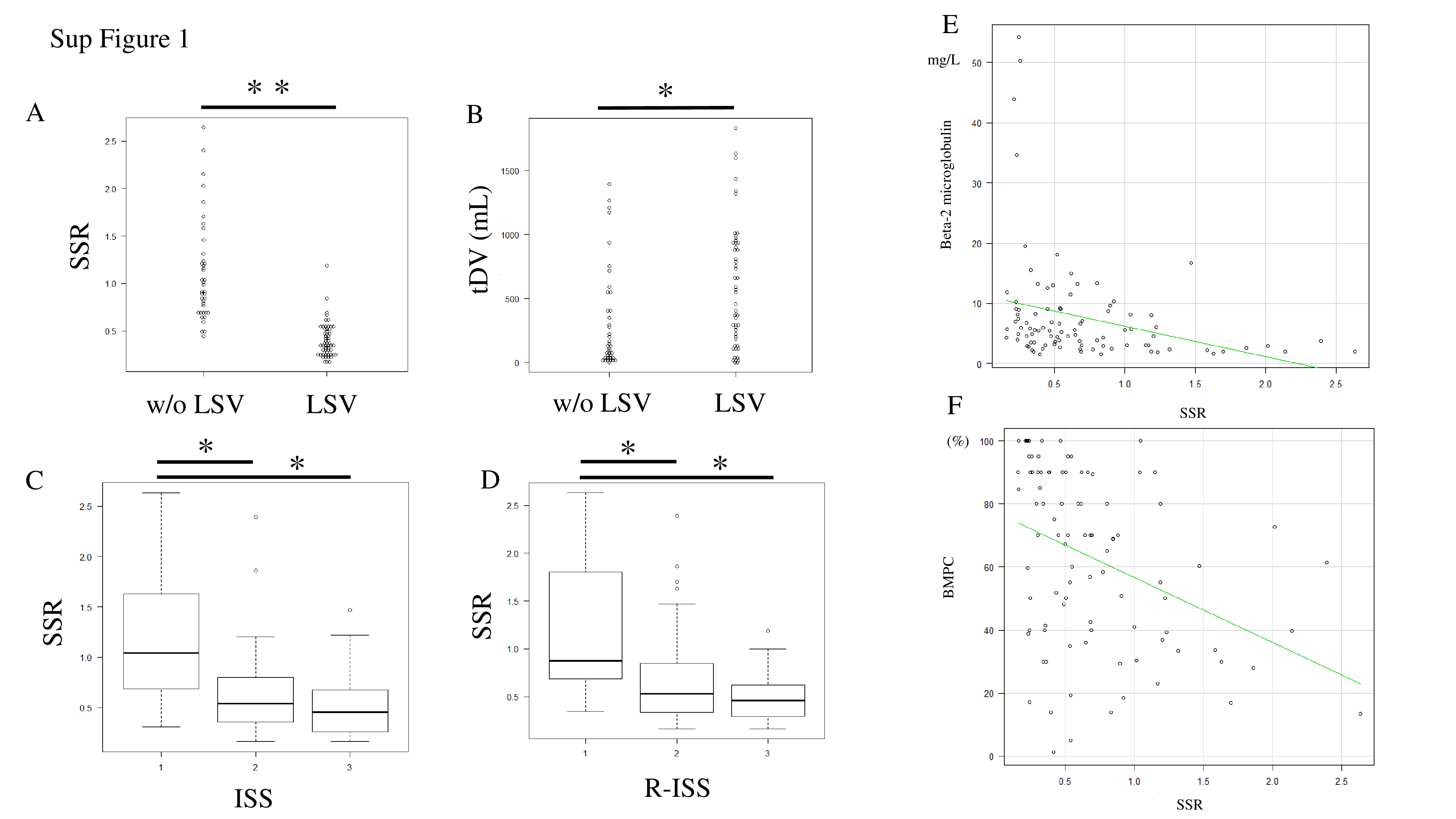

Supplement: Supplementary file 1 — Supplementary Information 1. [file 41598_2021_3496_MOESM1_ESM.tiff]

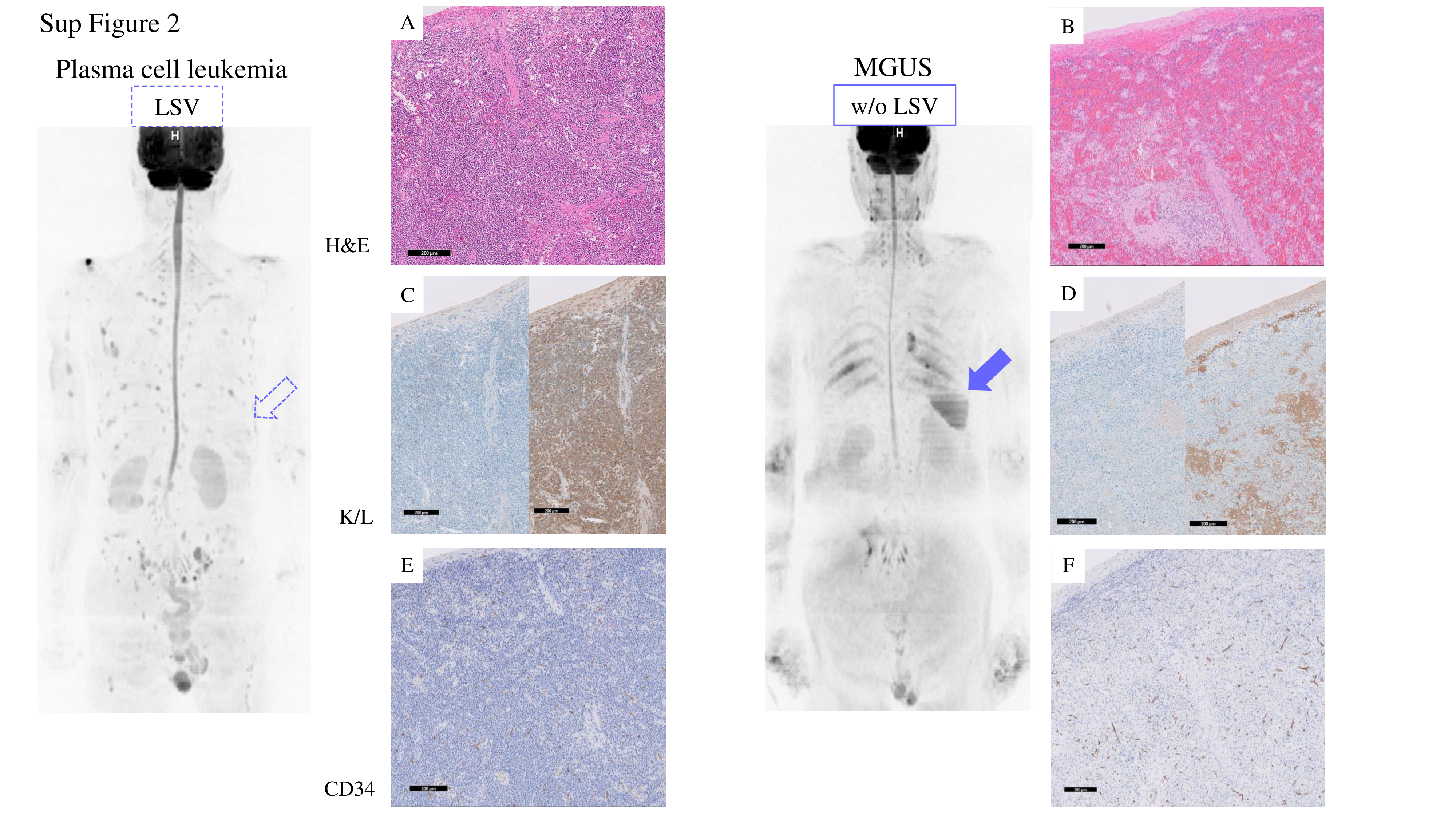

Supplement: Supplementary file 2 — Supplementary Information 2. [file 41598_2021_3496_MOESM2_ESM.tiff]

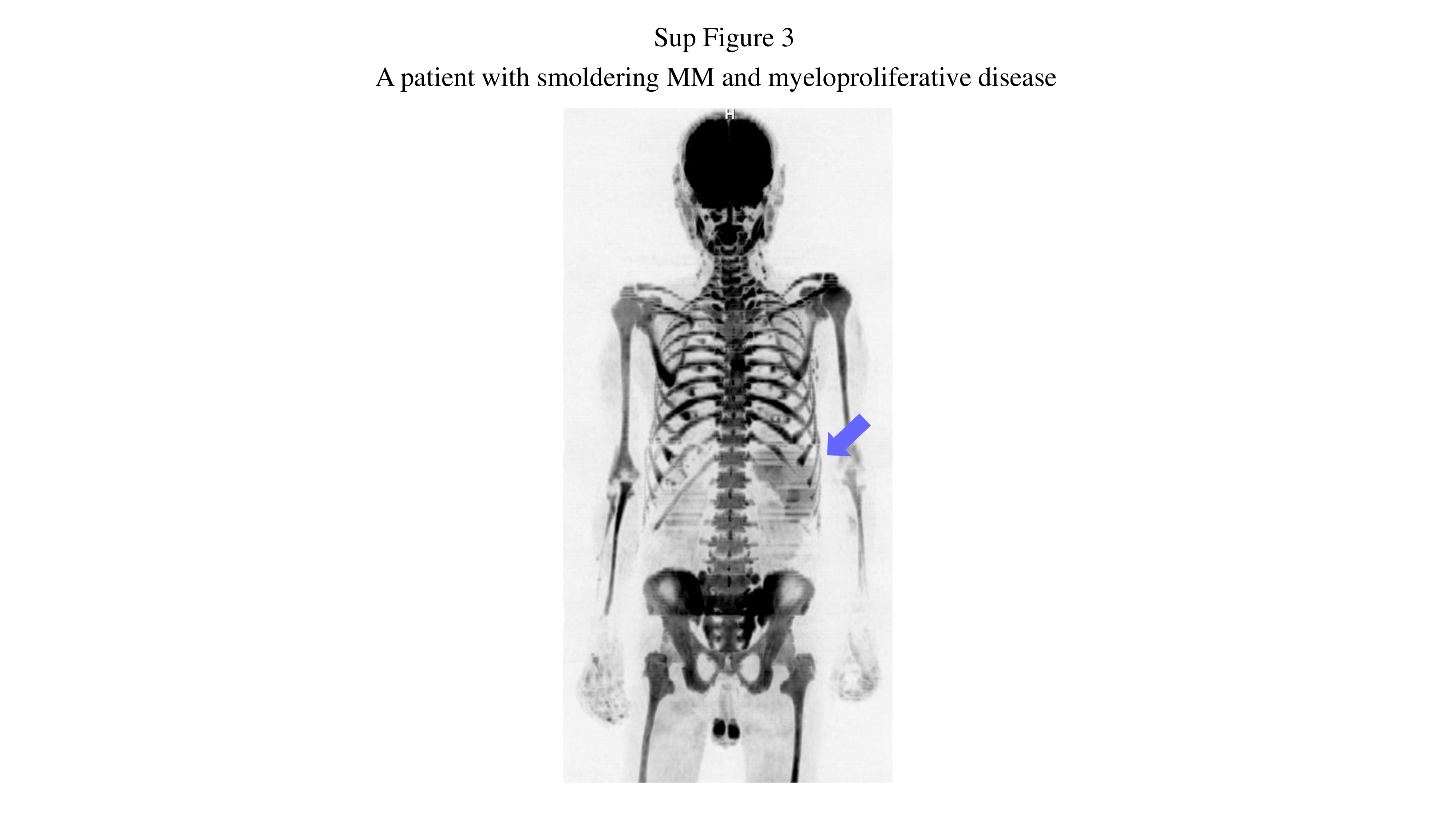

Supplement: Supplementary file 3 — Supplementary Information 3. [file 41598_2021_3496_MOESM3_ESM.tiff]
