# Supplementary material for: Association of loss of spleen visualization on whole-body diffusion-weighted imaging with prognosis and tumor burden in patients with multiple myeloma
Source: Sci Rep. 2021 Dec 14;11:23978. doi: 10.1038/s41598-021-03496-1 (PMC8671425; doi:10.1038/s41598-021-03496-1)
Supplement: Supplementary file 4 — Supplementary Information 4. [file 41598_2021_3496_MOESM4_ESM.docx]

**Supplementary Information**

**Supplemental Figure S1**

**Relationship between spleen signal and tumor burden**

**A.** Patients with loss of spleen visualization (LSV) at diagnosis have a lower median spleen-to-spinal cord ratio (SSR) than those without LSV at diagnosis (median SSR: 0.36 vs. 0.96, *p* < 0.001)

**B.** Patients with LSV at diagnosis have a higher median total diffusion volume (tDV) than those without LSV at diagnosis (median tDV: 540.2 vs. 137.0 mL, *p* = 0.003)

**C and D.** Patients with International staging system (ISS) stage I and R-ISS stage I have a higher SSR than those with ISS stage II-III (C) and R-ISS stage II-III (D). The median SSR in ISS stage I, II, and III were 1.04, 0.54, and 0.45, respectively. The median SSR in R-ISS stage I, II, and III were 0.88, 0.53, and 0.46, respectively.

**E and F.** The Spearman’s correlation coefficients between SSR and beta-2 microglobulin (E) and BMPC (F) were negatively correlated (beta-2 microglobulin; ɤs = -0.39, *P*<0.001, and BMPC; ɤs = -0.43, *P*<0.001).

Asterisks denote significant changes (*0.001 ≤ *p* < 0.01 and ** *p* < 0.001)

**Supplemental Figure S2**

**Histopathological findings**

Patient #1 had primary plasma cell leukemia and died due to disease progression. Patient # 1 had loss of spleen visualization (LSV) on the last whole-body diffusion-weighted imaging (WB-DWI) prior to death. Higher cellularity (**A**, hematoxylin & eosin [H&E]), and direct diffuse plasma cell infiltration in the spleen (**C**, the image on the left is immunohistochemistry for kappa [K], the image on the right is immunohistochemistry for lambda [L]) are observed. Patient #2 had monoclonal gammopathy of undetermined significance (MGUS) and systemic amyloidosis and died due to sepsis and arrhythmia. Patient # 2 did not have LSV on the last WB-DWI prior to death. Lower cellularity (**B**, H&E) and amyloid deposition in the spleen (**D**, amyloid is immunohistochemistry for lambda) are observed. Neither patient has evidence of extramedullary hematopoiesis (EMH) in the spleen. Only endothelial cells are positive for CD34 (**E** and **F**). The scale bar shows 200 micrometres.

w/o: without

**Supplemental Figure S3**

**Patient with smouldering multiple myeloma and myeloproliferative disorder**

This patient has smouldering multiple myeloma (MM) with a myeloproliferative disorder and is considered as having extramedullary hematopoiesis (EMH) in the spleen. The bone marrow is diffusely positive on whole-body diffusion-weighted imaging, though loss of spleen visualization (LSV) was not observed. These findings suggest that LSV requires a high myeloma burden rather than EMH in the spleen. The blue arrow indicates visible spleen.
